# Predation Risk Experienced by Tadpoles Shapes Personalities Before but Not After Metamorphosis

**DOI:** 10.1002/ece3.70532

**Published:** 2024-11-13

**Authors:** Barbara Płaskonka, Anna Zaborowska, Andrzej Mikulski, Barbara Pietrzak

**Affiliations:** ^1^ Department of Hydrobiology, Institute of Ecology, Faculty of Biology University of Warsaw Warsaw Poland; ^2^ Botanic Garden, Faculty of Biology University of Warsaw Warsaw Poland; ^3^ Faculty of Biology, Biological and Chemical Research Centre University of Warsaw Warsaw Poland

**Keywords:** animal personality, anti‐predatory response, anuran, behavioural repeatability, carryover effects, metamorphosis

## Abstract

Consistent inter‐individual differences in behaviour, that is, personalities, can emerge as a result of inter‐individual differences in ontogenetic experience, and predation risk is a potent one. As personalities develop over lifetime, however, they may also be broken by ontogenetic transitions of the individual. Here we first tested the hypothesis that consistent inter‐individual differences in larval behaviour arise under predation challenge, and are entangled with differences in body size. We then tested the hypothesis that adult behavioural type is related to body size rather than to larval behavioural phenotype. To test these hypotheses, we performed a longitudinal study following the development of about 50 moor frogs, *Rana arvalis*. We manipulated their larval and current environment, and recorded their behaviours repeatedly, under control conditions, invertebrate predators' chemical cues or in live predator presence. Partially in line with our predictions, the ontogenetic experience of predator presence led to personality emergence in tadpoles, yet their behaviour was not explained by their body size. This pattern was lost over metamorphosis. According to predictions, pre‐adult moor frog behaviour was affected by their body size—time to exit shelter was shorter in larger frogs—but neither by their behaviour as tadpoles nor by their larval environment, that is, tadpole predator‐exposure experience. Our results show that individual behavioural tendencies can be well decoupled between prior and post metamorphosis, which adds to the growing empirical evidence supporting adaptive decoupling hypothesis.

## Graphical abstract

We showed that ontogenetic experience of predator presence led to personality emergence in moor frog tadpoles, whose behaviour was yet not explained by their body size. We also showed this pattern was lost over metamorphosis, as juvenile frog behaviour was affected by their body size, but neither by their behaviour as tadpoles nor by their ontogenetic environment. Our results show that individual behavioural tendencies can be well decoupled between prior and post metamorphosis in an anuran.

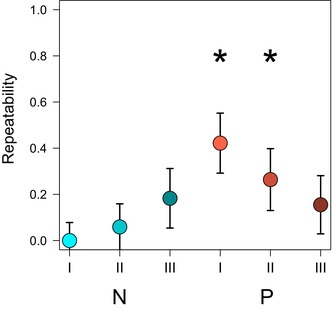

## 1 Introduction

It is now well recognised that individuals of a species differ and do not perceive or respond to their environment uniformly (Cabrera, Nilsson, and Griffen 2021; Greven et al. 2019; Sih et al. 2015). Consistent inter‐individual differences in behaviour, that is, personalities, arise via both developmental stochasticity (Laskowski, Chang, et al. 2022; Laskowski, Bierbach, et al. 2022; Takagi and Benton 2020) and genotype–environment interactions. Moreover, the environment may act both before (via transgenerational effects) and throughout the lifetime of an individual (ontogenetic and current environmental context; Laskowski, Chang, et al. 2022; Laskowski, Bierbach, et al. 2022; Tariel, Plénet, and Luquet 2020). Decline of behavioural plasticity over lifetime (Polverino et al. 2016) via experience and learning may be part of the process of personality emergence (Cooke et al. 2021). Personalities are often detected as the repeatability of behaviour, that is, the significant proportion of inter‐individual variance relative to the total phenotypic variance in a population, for repeated measures of the same behaviour (Dingemanse et al. 2010).

### 1.1 Personalities and Predation Risk Experience

Experience with predators is a fitness‐related environmental context and it affects both the average behaviour of individuals and how individuals differ from each other (Bucklaew and Dochtermann 2021). Predation risk is also one of those environmental contexts under which personalities are observed to emerge (Abbey‐Lee and Dingemanse 2019; Dawidowicz et al. 2023; Heuschele et al. 2020; Toscano et al. 2014). It has been proposed that this repeatability emerges because the intra‐individual variation introduced by other environmental contexts is reduced under predation risk, due to the fitness‐related potency of the predation context (Toscano et al. 2014). It might also come about from a kind of conditioning and reinforcement of the first exhibited response if it was successful, that is, the animal has survived the perceived threat and continues to express this first‐response behaviour.

The emergence of personalities under predation risk might also be a result of state–behaviour correlations and positive feedbacks. An example of behaviour correlating with state (or body condition) is when larger individuals are less vulnerable to predation and thus behave more boldly. A state–behaviour positive feedback is when the traits reinforce reciprocally, for example, larger, less vulnerable individuals train themselves in bold foraging behaviours and thus acquire more resources and grow even larger. Yet still, theory predicts and awaits empirical evidence of inter‐individual variation emerging from positive feedback loops between states and behaviour (Ehlman, Scherer, and Wolf 2022; Sih et al. 2015). Some evidence has come so far, for example, from human psychology studies (Layous et al. 2017) and animal social interactions, for example, sociality found in a positive feedback loop with social competence (Taborsky 2021) or for experience combined with other effects in birds (Cooke et al. 2021).

Variation in several suggested intrinsic state variables, including body size, body condition, metabolic rates and hormone levels, explains a small, yet significant share of the inter‐individual variation in behaviour across studies (Niemelä and Dingemanse 2018). Body size is of expected importance under the circumstances of predation threat. Indeed, one of the proposed mechanisms generating feedbacks between state and behaviour is state‐dependent safety. The assumption here is that individuals with higher state, like better condition or larger size, face lower risk of predation while being bold (Luttbeg and Sih 2010; Sih et al. 2015). The expected positive feedback stems from that the individuals in better condition behave more boldly, thus acquiring more resources, thereby further increasing in condition.

### 1.2 Personalities Over Metamorphosis

Finally, experience may not matter if during development the animal changes its environment or changes itself completely. Most animals go through distinct discrete phases over their ontogeny, a form of metamorphosis, with rapid transformations, which include morphology and physiology (Cabrera, Nilsson, and Griffen 2021; Moran 1994). Thus, understanding if and how personalities arise and are maintained through such transformations is of key importance (Wilson and Krause 2012a). These transformations are often coupled with the shift of the habitat or ecological niche (e.g., aquatic to terrestrial, herbivore to carnivore), and are associated with changes in the selective pressures acting on individuals (Stoks and Crdoba‐Aguilar 2012). Naturally, behaviours change or new ones arise according to new challenges (Kaiser, Merckx, and Van Dyck 2018; Moran 1994; Wilson and Krause 2012b).

Hence, according to the adaptive decoupling hypothesis (Haldane 1932; Moran 1994), individual behavioural tendencies or personality, seen before a major transition such as metamorphosis, should not persist after it (Bell and Stamps 2004; Sih, Bell, and Johnson 2004; Sih et al. 2004). Yet, empirical studies on holometabolous insects bring mixed results (Monceau et al. 2017; Müller and Müller 2015; Wexler et al. 2016; Rodrigues et al. 2016; Brodin 2009). There appears to be no single pattern applying to all species undergoing complete metamorphosis, but instead, personality retention could be influenced by factors other than the juvenile to adult niche shift only (Kaiser, Merckx, and Van Dyck 2018).

Amphibians, as the only vertebrates undergoing metamorphosis, have also been studied for maintaining their behavioural type through this developmental process. Anurans, undergoing the most drastic morphological changes, are a good model here, and especially interesting among them are those species in which adults are terrestrial and only return to water bodies for reproduction, such as moor frogs or most poison frogs (Loman 1978; Ringler et al. 2013, 2018; Sjögren‐Gulve 1998). Thus, ecological niches of tadpoles (aquatic) and adults (terrestrial) differ considerably, which might favour decoupled personality traits across development (Bégué et al. 2023). Table 1 summarises research on the consistency of behaviour before and after metamorphosis in amphibians, most performed in a single genetic or developmental context, and some of which have recorded persistence of the behavioural type through the transformation. Results are difficult to compare due to different definitions and behavioural tests used to assess personality traits, different sample sizes potentially resulting in different statistical power to detect differences and different statistical tools used to assess consistency across metamorphosis (Table 1).

**TABLE 1:** Behavioural consistency over metamorphosis in amphibians: four anurans and a salamander.

Species	Shift	Behavioural parameter		*N*	Timespan	How consistency tested	Reference
*Allobates femoralis*	Full/full	Distance travelled in OFT	E	17	18/15/12	Models with ‘stage’ as fixed effect + correlations	Bégué et al. (2023)
		**Crossing counts to/from refuge**	**E**				
		**Probability of exiting refuge**	**B**				
		**Latency to exit refuge**	**B**				
		**Time in open space**	**B**				
*Rana arvalis*	Full/full	Latency to exit refuge	B	30	NA	Models with ‘stage’ as fixed effect + correlations (different contexts)	Cortazar‐Chinarro et al. (2023)
*Rana arvalis*	Full/full	Time in open space	B	49	14/10/3	Correlations (different contexts)	This study
		Immobility time	A				
*Rana ridibunda*	Full/partial	**Composite, PCA collapsed traits**	**A/E**	45	1–2/+8/1	Correlations	Wilson and Krause (2012b)
*Rana temporaria*	Full/partial	Space use (area covered) in OFT	E	(80)	12/NA/NA	Repeatability (different contexts)	Brodin et al. (2013)
		Latency to exit refuge	B				
*Ambystoma maculatum*	Partial/partial	**Composite, PCA collapsed traits**	**~B**	40	1/15/1	Correlations among behaviours	Koenig and Ousterhout (2018)

*Note:* Shift: morphological (metamorphosis)/ecological (niche). Larvae and juveniles were tested, and where their personality parameters were deemed consistent they are indicated in bold. Personality dimension assigned by the authors: A, activity; B, boldness; E, exploration; OFT, open field test; *N*, number of individuals tested across metamorphosis; Timespan, average number of days elapsed between assays (first and last larval/last larval and first juvenile/first and last juvenile); + denotes only days after metamorphosis. Different contexts refer to geographic, habitat type or developmental heterogeneity of the studied population.

Here, we first tested the hypothesis that (H1) consistent inter‐individual differences in behaviour arise (i) under predation challenge and (ii) via body size–behaviour feedbacks. We expected (i) boldness and activity of the tadpoles exposed to predator cues to be more repeatable than those of control tadpoles. We also expected (ii) body size to explain partly this inter‐individual variance. Second, we tested the hypothesis that (H2) adult behavioural type is related to state (body size) rather than to larval behavioural phenotype. To test these hypotheses, we performed a longitudinal study following the development of several dozens of moor frogs, manipulating their environment and recording their behaviours repeatedly.

## 2 Methods

### 2.1 Study Organism and Site

The moor frog (*Rana arvalis*) was chosen for the study for several reasons. It is one of the most abundant lowland frog species living in central‐eastern Europe. In north‐eastern Poland, it reproduces in wetlands and water reservoirs in late March/early April and hibernates on land, burrowing into the soil. Hence, adults are easily caught during their spring migration to water bodies. Females can lay between 1000 and 2000 eggs, making it possible to use multiple offspring of a single parent in an experiment (Hettyey, Herczeg, and Hoi 2009). Finally, the particular population chosen here was studied for several years, having predictable abundances and phenology.

Since in the experiments we included both larval and adult forms, the research covered the period from the beginning of March to the first half of August 2019. It was carried out in the area of the Masurian Landscape Park, at one of the ponds located at the fallow fields (53°49′14.0″ N, 21°39′27.0″ E) next to the Masurian Centre for Biodiversity, Research and Education KUMAK in Urwitałt, Poland, where the experiments were performed.

The research was permitted by the National Ethics Committee on Animal Experiments and, since the moor frog is under protection in Poland, also permitted by the Regional Director for Environmental Protection.

### 2.2 Experimental Animals

Five adult males and five adult females caught while migrating to the breeding ground or directly at the pond were paired. Each pair was allowed to mate in a separate plastic container (41 × 34 × 23 cm) filled with water and plants from the pond, which was placed in the pond to ensure conditions similar to those prevailing there. After spawning, the adults were soon released, and the eggs were carefully transported to the laboratory at the research centre in the containers where they were laid. The water in the containers was repeatedly refreshed with pond water that had previously been conditioned and aerated in a separate container for at least 48 h (conditioned thereafter) to allow the degradation of the chemical cues of predation (kairomone) (Van Buskirk et al. 2014). After hatching, the tadpoles were kept in groups in the same containers, with submerged macrophytes and detritus taken from their pond of origin. Macrophytes and detritus were rinsed thoroughly and conditioned in a separate container in pond water before use. This material provided substratum and food to the developing larvae and together with the social environment ensured a minimum of early experience gained through rearing in a semi‐natural environment. This is important to allow valid comparisons between studies of wild and laboratory test populations (Stamps and Groothuis 2010; Wilson and Krause 2012b), while providing kairomone naïvety for control animals. Again, the water was exchanged weekly with fresh conditioned pond water.

Approximately upon reaching the stage 37 (Gosner 1960), the tadpoles were randomly taken from the containers, randomly assigned to one of the two larval environments, see next, behaviourally tested and placed individually in separate 1.5 L transparent plastic rearing containers (19 × 12 × 10 cm) where they stayed through metamorphosis until the end of the experiments. The boxes contained 0.75 L conditioned, aerated pond water, refreshed 3–4 times per week and plants from the pond, providing food and refuge, exchanged and cleaned weekly. The two rearing, that is, larval environment, treatments were: N—control, no predator and P—predator cues. Control tadpoles (N) had the water refreshed with at least 48‐h conditioned pond water mixed with well water in a ratio of 1:1, while predator‐exposed (P) tadpoles had the water refreshed with the same water additionally enriched with predator cues. The predator‐cue water was obtained by keeping invertebrate predators in the conditioned pond water mixed with well water (1:1). The predators were beetles, Dytiscidae larvae and Hydrophilidae both larvae and adults, and dragonflies, Libellulidae larvae (in ground majority of the species: *Dytiscus marginalis*, *Acilius sulcatus*, *Cybister lateralimarginalis*, *Sympetrum vulgatum*, *Leucorrhinia albifrons* and *Libellula quadrimaculata*). Each predator was housed individually in a water‐filled container (100 mL) with some living plant shoot or plant debris. On the day of the experiment, the water from the containers was gently poured through a mesh, pooled and topped up with 48‐h conditioned pond water (1:9). The predators were gently rinsed with 100 ml pond:well water mix back to their containers and fed live fish food, mainly *Tubifex* larvae. The tadpoles were fed daily with algae and multi‐ingredient aquarium foods supplied ad libitum.

After metamorphosis, the froglets were again kept in separate 1.5 L transparent plastic rearing containers (19 × 12 × 10 cm). We added floral foam covered with moss to the container to provide a terrestrial environment. The froglets were fed daily with flightless *Drosophila melanogaster* and *D. hydei*. For the whole duration of the study, the rearing boxes were covered with an openwork lid keeping away potential predators. They were placed in a daily randomised arrangement, outside, among trees, on a low platform, under a dark, waterproof artificial roof. On particularly sunny days, they were additionally shaded from the sides with camouflage net. After final behavioural testing, all froglets, as well as any tadpoles that have not been used in experiments, were released at the site where the adults were captured.

### 2.3 Behavioural Tests

#### 2.3.1 Tadpoles

**FIGURE 1:**
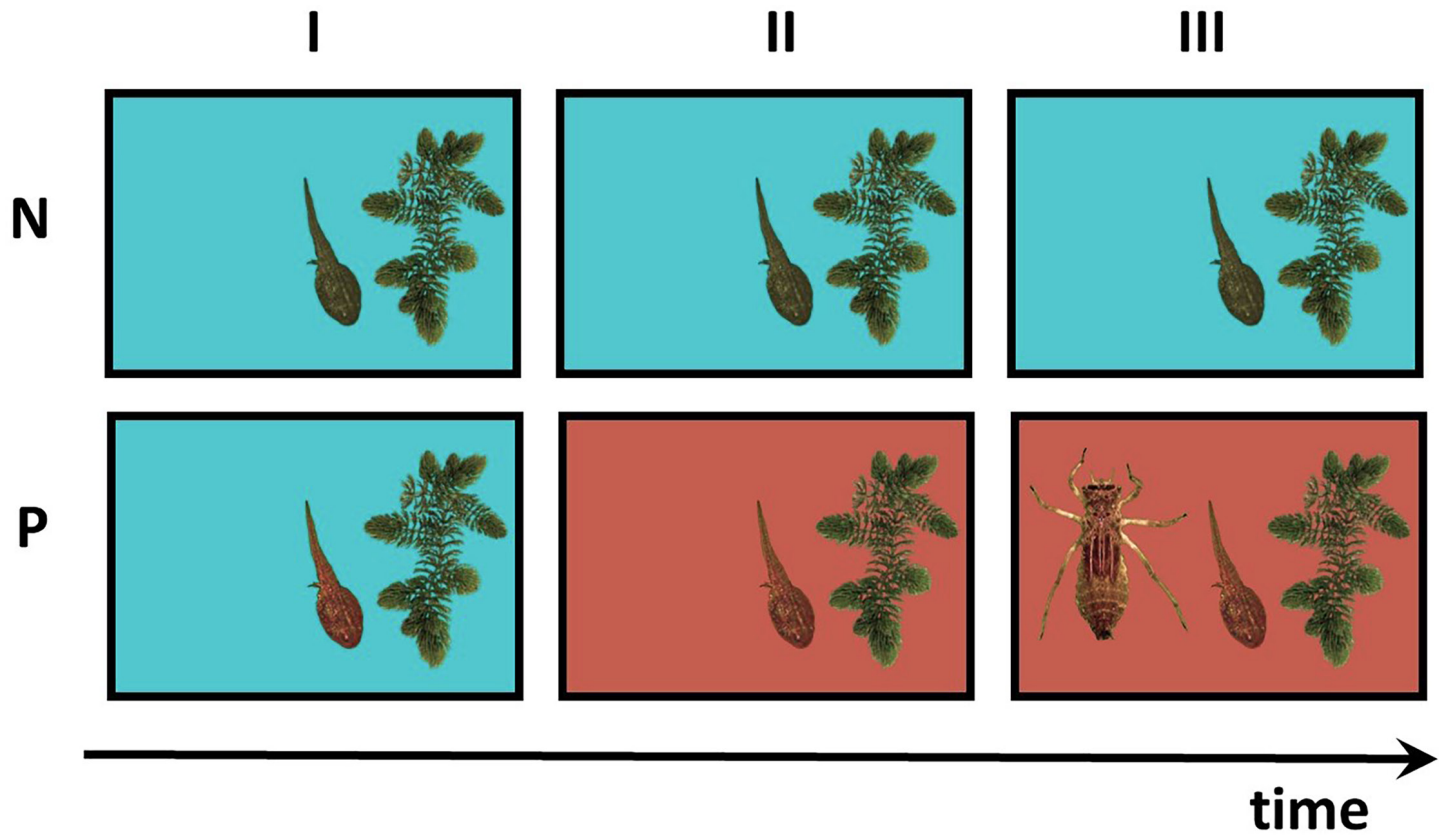
Illustrative diagram of a behavioural assay divided into three ‘current environment’ phases: I—initial, II—predator cue (red area signifies the presence of the chemical cues of predators) and III—live predator (insect larva symbol) presented for two tadpoles reared in different ‘larval environments’: no predator (upper panels, N, greenish tadpole) or predator exposed (lower panels, P, reddish tadpole). Organisms not to scale.

We used eight opaque flat‐bottomed plastic containers (24 × 39 × 7.5 cm) as experimental arenas, placed on an evenly shaded table in a randomised arrangement. We poured 2.5 L of conditioned pond water into each container and added a few short shoots of *Ceratophyllum demersum* to one side of the arena to create a semi‐natural shelter. Then we gently placed one, randomly chosen tadpole into the arena and started recording the arena with a Sony α7 camera placed 1.5 m above the arenas. At one time we recorded eight arenas (four N and four P tadpoles). A behavioural assay was divided into three phases (current environmental conditions) lasting 15 min each (45 min together) and during each, we made a video recording of the tadpoles' behaviour. These three phases were: (I) initial phase—the tadpoles were acclimated in the arena and their behaviour recorded, while there was only control water and plants in all containers. Thus, individuals of different ontogenetic experience (larval environment N or P) were here observed under the same control conditions; (II) predator‐cue phase—the main phase of the assay which began after adding: 0.5 L of control, conditioned pond water to four arenas with tadpoles from the control treatment (N) and 0.5 L of predator‐cue water to the remaining four arenas (P). Thus individuals of different ontogenetic experience were observed during their defining control or predator exposition and (III) live predator phase—when the P tadpoles were exposed to a single live predatory insect larva after it was gently placed in the open area of each P experimental arena. This phase presented nothing new to the N tadpoles, while providing the P tadpoles with an experience more resembling a natural one, a real encounter involving visual and mechanical cues. The predators came randomly from among the same containers from which we obtained the chemical cues of predators. Thus, P tadpoles could be exposed to predators of different species over the three assays repeated on different days, but a single P tadpole was exposed to a single predator at a time. The control arenas remained unchanged in phase III (Figure 1). Thus, N tadpoles remained naïve throughout the experiment, that is, at no point in life were they exposed to either chemical cues of or live predator itself. After phase III, each tadpole was photographed on a gridded Petri dish to determine its size [head–tip to tail–base length; as body length was a good predictor of body mass (data not shown), we kept to these measurements] and placed back in its rearing box. The experiment was repeated (three assays) at three different dates for each tadpole, all done before the animal reached stage 42 (spanning over 16 ± 4 days, mean ± SD, not differing between N and P tadpoles, Student's *t* test: *t* = 0.53, df = 46, *p* = 0.6; *n* = 49).

#### 2.3.2 Juvenile Frogs

The first froglet assay was performed 2 or 3 days after the completion of metamorphosis, that is, after the tail was resorbed and the animal spent most time on land (10 ± 3 days, mean ± SD, after the last tadpole assay, not differing between N and P tadpoles, Student's *t* test: *t* = 0.333, df = 44.3, *p* = 0.74). We used eight arenas as before, filled with soil. On the one side of each arena we placed a paper cup (diameter of 9.5 cm and a height of 11.5 cm) upside down as a refuge. In all cups we cut a small hole (4 × 4 cm) at the edge on the ground. One froglet was placed under each cup. We recorded the time after which the animal came out through the cut hole. If the frog did not leave the refuge for 15 min after being placed under the cup, it was recorded that it did not come out at all (censored cases). The test was repeated (three assays) at three different dates for each froglet (spanning over 3 ± 1 days, mean ± SD, not differing between N and P animals, Student's *t* test: *t* = −1.55, df = 44.8, *p* = 0.128).

### 2.4 Recording Analysis

In this study, we analysed the recordings of the 49 tadpoles (5–14 from each family; 24 N and 25 P) that had gone through successful metamorphosis by the end of our experiment. To do the analysis, we used EthoVision XT software (Noldus). We began analysing the video 1 min after the tadpole was placed into the arena (phase I), control water or predator‐cue water was poured in (II) or the live predators were placed in the arenas of P tadpoles (III). We analysed the subsequent 10 min of each recording. Data were obtained in the form of tadpole position coordinates and parameters calculated by the software: time spent by tadpoles among plants, at the wall of the arena or in the open space of the arena, based on the plants area (about 1:3 of the arena space) and walls area (less than 2 cm from the sides, earlier observations suggested this zone may be perceived by the tadpoles differently than open space of the arena) manually marked before analysis. From the coordinates we calculated the time the tadpole remained motionless and the tadpole's movement speeds. Data were manually curated and readings generating speeds over 20 cm/s were removed (Huey 1980).

### 2.5 Statistical Analysis

We analysed the following behavioural parameters for each animal:

1. Tadpole boldness, proxied with microhabitat selection: The main parameter here was the proportion of time spent in the open—calculated as the time spent by the tadpole outside the plant area and the walls area over the total observation time. Two auxiliary parameters were the proportion of time spent among plants—the time spent by the tadpole in the plants area over the total observation time, and the proportion of time spent at the walls—the time spent by the tadpole in the walls area over the total observation time.
2. Tadpole activity: The main parameter here was the proportion of time spent in motion—calculated on the basis of position coordinates of the tadpole in the arena. The number of readings without repositioning was divided by the total number of position reads. Two auxiliary parameters were median and third quartile, respectively, of instantaneous velocities—calculated on the basis of changes in the position of the tadpole in the container between successive readings.
3. Froglet boldness: The time taken by the frog to come out of its hiding place.

We analysed the effects of the parental pair, larval environment (N or P), current environment (phase I, II or III) and of the current individual body size (body length) on the above‐mentioned parameters. To allow for correlation of errors resulting from repeated recordings for individual tadpoles and for unequal variances, we fitted a linear model using generalised least squares (GLS) by maximising the restricted log likelihood (nlme package, Pinheiro et al. 2023). Repeated measurements for individual tadpoles were considered in the error structure of the model and type III analysis of variance (ANOVA) table was calculated for the model using Anova function of car package (Fox and Weisberg 2019). As the effects of body size and family of origin (parental pair) were not significant, neither as main effects nor in interactions, for any of the tested tadpole behavioural parameters, we dropped them from the final models. Also, we dropped the interaction between the effects of ontogenetic and current environment where the no‐interaction model was significantly better (lower AIC, *p* < 0.05 in model comparison with anova R function), that is, for tadpole boldness parameters. Pairwise comparisons between group levels with corrections for multiple testing were calculated using pairwise Wilcoxon rank sum tests with Holm correction.

To estimate the relative contribution of differences between individuals and within a single individual to overall variation, we used the R package rptR (Nakagawa and Schielzeth 2010; Stoffel, Nakagawa, and Schielzeth 2017). We estimated among‐individual variance (Vi), intra‐individual variance (Vr) and repeatability (R) using the linear mixed models (LMM) method (rptGaussian function). To meet the method assumptions, we transformed the data. We used the square‐transformed times spent in the open (tadpole boldness) and square‐root‐transformed times immobile (tadpole activity) to fit LMM independently for different treatment data. We based the estimation of 95% confidence intervals (CI) and *p* values on 1000 bootstrapping runs and 1000 permutations respectively.

To control if intra‐individual variance was tied to the timespan over which the tests were performed for individual tadpoles we performed Pearson's product–moment correlation on the variance from all nine measurements for each tadpole and the time between the first and last tests for the given individual (cor.test R function). Tadpole growth rate was estimated using their length on the date of the first and third tests (full size data available for 38 tadpoles) and timespan between the dates.

To test for differences in time to frog emergence from the hiding place, mixed effects Cox proportional hazards model (coxme package and function, Therneau 2022) was fitted to time to emergence data using tadpole larval environment and frog current body length as covariate. To test for associations between behaviour in the larval and pre‐adult stage, Cox proportional hazards model (coxph in *survival* package, Therneau 2023) was fitted to individual median values of frog time to emergence data using individual tadpole median values of the behavioural parameters measured as covariates. Wald statistic *z* was used to evaluate whether the coefficients of the variables tested were statistically significantly different from 0. Also, correlations in behaviour across metamorphosis and between behaviour and state variables were calculated (Pearson's product–moment correlation, cor.test R function). All calculations were performed using the R language and environment (R Core Team 2023).

## 3 Results

**FIGURE A1:**
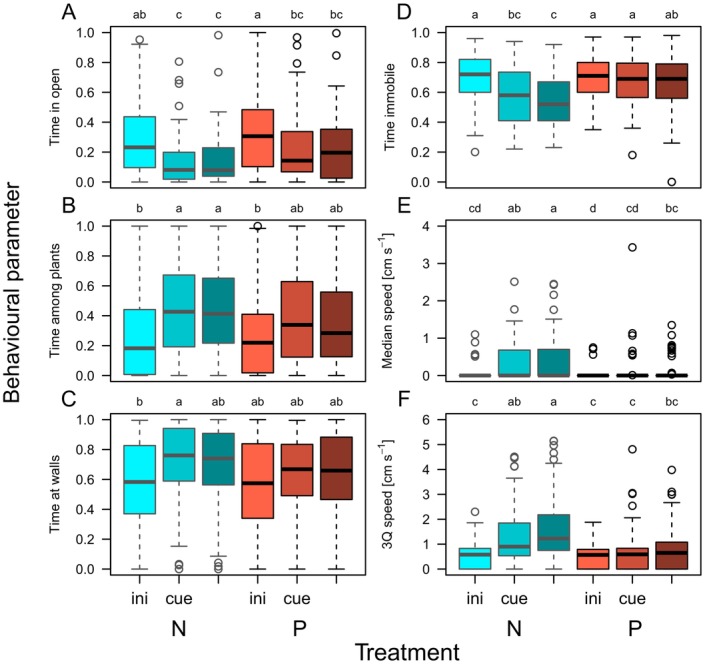
Boldness (A–C) and activity (D–F) of control, not‐exposed (N) and predator‐exposed (P) tadpoles in the three phases of the experiment, that is, under three current environment conditions: initial (I), predator cue introduced to P tadpoles (II) or live predator present in their arena (III). Median and quartiles.

Both ontogenetic and current environmental conditions affected tadpole boldness and activity, which were weakly correlated (Figure A1, Table A1). Predator‐exposed tadpoles stayed more in the open area of the arena and remained inactive after the addition of the predator‐cue medium (Figure A1, Table A1).

**FIGURE 2:**
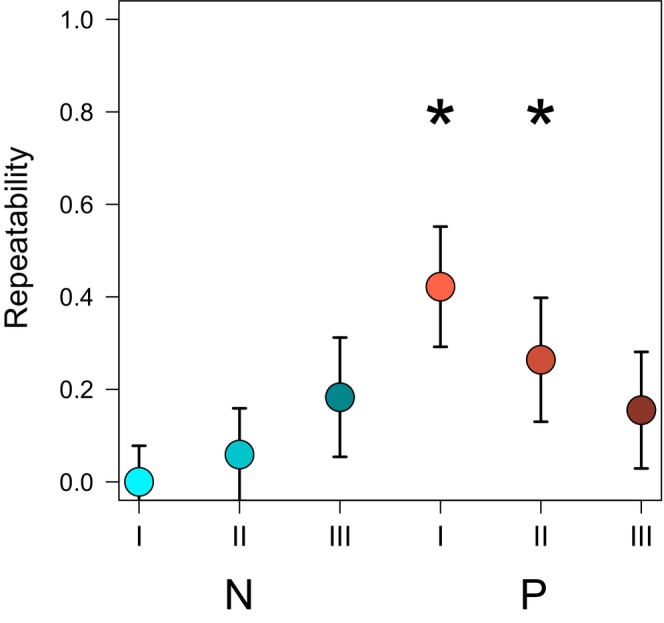
LMM‐based estimates of repeatability of control (N, blue symbols) and exposed (P, red symbols) tadpole activity throughout the three phases of the experiments, that is, current environments (I–III). Whiskers denote standard errors. Asterisks mark values significantly different from zero at *p* < 0.05 (based on permutation tests).

Tadpoles receiving predator cues (P) were significantly repeatable in their activity, proxied as the time spent immobile, in the initial (I|P: *R* = 0.42) and predator‐cue phases (II|P: *R* = 0.26, Figure 2, Table A2), yet, the repeatability was lost after the specific live predator was added. Control tadpoles behaviour (N) was not repeatable under any of the current environment phases (Figure 2, Table A2). Also, tadpole boldness, proxied as the time they spent in the open, was not repeatable under any ontogenetic or current environment (Table A3). The repeatable activity was not correlated with the current tadpole size (I|P: *t* = −1.31, df = 60, *p* = 0.19, II|P: *t* = 0.21, df = 59, *p* = 0.83).

Neither for activity nor for boldness the overall intra‐individual variance correlated with the timespan over which the tests were performed for individual tadpoles (Pearson's product–moment correlation, |cor| < 0.18, *p* > 0.22). Mean tadpole growth rate over their testing period was 0.12 ± 0.08 mm/day (mean ± SD). N and P tadpoles did not differ in growth rate (Student's *t* test: *t* = 0.62, df = 36.7, *p* = 0.54), which was not correlated with either boldness or activity (|cor| < 0.2, *p* > 0.22).

**FIGURE 3:**
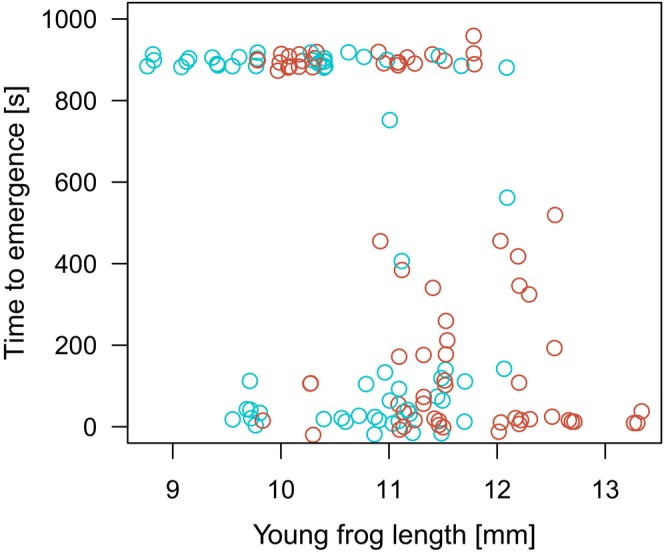
Time to froglet emergence from the refuge. Blue—N, red—P tadpoles. Data points jittered for figure clarity. Points jittered around 900 s are censored cases, that is, frogs have not emerged within 15 min.

**FIGURE 4:**
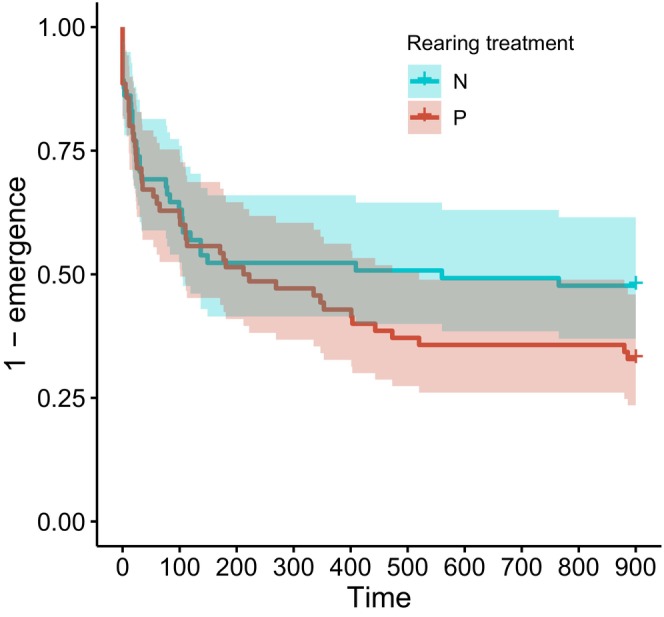
Share of the froglets that remained in the refuge with elapsed time (s) according to their larval environment (N or P). Shaded area—confidence intervals.

Larger froglets left their hiding place faster (Cox mixed effects model, effect of body length: HR = 2.27, *z* = 4.76, *p* < 0.0001) (Figure 3). Time to emergence of the froglet from the hiding place was not explained by the tadpole larval environment (N/P treatment) (HR = 0.69, *z* = −1.13, *p* = 0.26) (Figure 4). None of the tadpole behavioural parameters, taken as mean or median of the nine measurements, correlated with frog behaviour, taken as mean, median or sum of the three measurements (*p* > 0.15).

## 4 Discussion

This study shows that individual behavioural tendencies can be well decoupled between prior and post metamorphosis, which adds to the growing empirical evidence supporting adaptive decoupling hypothesis (Haldane 1932; Moran 1994). Partially in line with our predictions (H1), the ontogenetic experience of predator presence led to personality emergence in tadpoles. Yet, their behaviour was not explained by their body size. This pattern was lost over metamorphosis. According to predictions (H2), pre‐adult moor frog behaviour was affected by their body size—time to emergence was shorter in larger frogs—and it was not affected by their behaviour as tadpoles nor by their larval environment, that is, tadpole predation–exposition experience.

### 4.1 Consistent Inter‐Individual Differences in Behaviour Arise Under Predation Challenge (ad. H1.i)

Before metamorphosis, exposure to predation threat induced repeatability of tadpole activity under two of the three tested contexts. Exposed tadpoles (P) were consistent in the time they spent immobile in the initial (I) and main (II) phase of the assays, that is, before adding a live predator. Meanwhile, this consistency was not observed for tadpole boldness, proxied as the time spent in the open area of the arena. Thus, this result contrasts with the observation made in many taxa that activity is rather not a repeatable trait in general (Bell, Hankison, and Laskowski 2009). Yet, importantly, this repeatability (i.e., personality) was recorded here in two environmental contexts that elicited different behaviours, and over a relatively long period of time at the order of 2 weeks. Here we held the animals in isolation in semi‐laboratory conditions for these long periods between behavioural measurements. We are aware this can impede learning and development dependent on field conditions or experiences, which may drive behavioural change in the field (Archard and Braithwaite 2010; Toscano et al. 2014). Yet again, in unexposed (N) tadpoles repeatability was not recorded in our experiment. And it was indeed in mobility that the differences between no predation and predation treatments were seen (Figure A1).

These results corroborate some of the earlier findings on tadpole personalities. Agile frog, *Rana dalmatina*, tadpoles reared alone and without predator cues showed no evidence for repeatability (Urszán et al. 2015). Also, competition generated repeatability in tungara frogs (*Engystomops pustulosus*, Beyts et al. 2023). Yet, in Italian tree frog (*Hyla intermedia*) behavioural repeatability was found higher in predator‐naïve than in predator‐experienced tadpoles (Castellano and Friard 2021).

Our results showing personality emerging under predation threat stay in concordance with a handful of studies on other vertebrates and some crustaceans. Repeatability of activity‐related traits was higher under predation risk, than under none, in a planktonic copepod, though it declined with the length of the period considered (Heuschele et al. 2020, but see Heuschele et al. 2017 for another threat). Also in *Daphnia* repeatability was recorded under predation threat, but not in its absence, for a boldness and activity‐related trait (refuge use via diel vertical migration) and was suggested as a risk‐spreading strategy (Dawidowicz et al. 2023). Similarly, the repeatability of crab refuge use was higher under predation risk than in its absence (Toscano et al. 2014). Finally, while there was no difference in trait population means, repeatability of a phenological trait was lower under lower predation risk, while repeatability of exploration was not affected in a bird (Abbey‐Lee and Dingemanse 2019).

Interestingly, behavioural repeatability of the exposed tadpoles in our study was lost in the third phase of the assays (III), when specific predators were introduced into the arenas, clearly confirming that repeatability can be manipulated in experiments by given contexts (Niemelä and Dingemanse 2017). This loss of repeatability here can be due to the fact that in the direct exposition the tadpoles were facing a different, random from the mixed pool, live predator on each trial. Specific danger may elicit specific responses and different hunting modes demand different prey tactics to maximise survival. Through our experiment, most P tadpoles were exposed to different live predators in subsequent assays. It also seems that the presence of a live beetle larva made the tadpoles avoid the plant ‘littoral’ more than the presence of a dragonfly larva did (Appendix 1). Hence, possibly time in open area, versus time in plant cover, may be here not associated with boldness but its reverse, shyness.

Repeatability here was seen before this direct and particular exposition, that is, it was seen under a taxon‐broad mix of predator cues which reflected the invertebrate predator pool in the tadpole habitat of origin. Repeatable response under multiple‐predator threat, when there is no optimal tactic, could root in the simple mechanism of repeating the initially adopted behaviour that appeared successful, as the animal remained alive. Thus, exposed tadpoles were all subject to a certain, common conditioning, while control tadpoles were not. This repeatability under predation challenge could also emerge from a state‐behaviour feedback mechanism, if it was linked and looped with some physiological response, for example, cortisol levels. We have not studied physiological states here, though.

### 4.2 Consistent Inter‐Individual Differences in Behaviour Do Not Arise via Body Size‐Behaviour Feedbacks (ad. H1.ii)

Yet, neither overall tadpole behaviour nor the repeatable behaviour were explained by another state variable, tadpole body size, suggesting the observed repeatability has not emerged via size‐dependent feedback loop, as was expected. Size should be an important phenotypic trait affecting survival in face of predation. Larger tadpoles may attain ‘size refuge’ against some of the invertebrate predators (Koenig and Ousterhout 2018), used in this study. Also, optimal behaviour under this threat could be expected to be different in smaller and bigger individuals. Yet, we did not observe that. One explanation might be the isolation and lack of reference for assessing one's own size. It was thus not the trait that generated repeatability in the tadpoles. In fact, tadpoles grew between trials so size itself changed while behaviour remained constant to some extent.

Earlier studies do not provide a clear picture on the relationship between tadpole behaviour and body size. Tadpole body length correlated with proportion of time spent in open area, total activity and latency to first movement and to resume activity in lake frog, *Rana ridibunda* (Wilson and Krause 2012b). Meanwhile, weight did not affect boldness of the common frog, *R. temporaria*, tadpoles, but heavier tadpoles had a higher exploration index (Brodin et al. 2013). Recently, Bégué et al. (2023) found that exploration‐ and boldness‐related behaviours were correlated with growth speed in brilliant‐thighed poison frog, *Allobates femoralis*, tadpoles. Very bold and explorative individuals took fewer days until metamorphosis compared to very shy and non‐explorative ones. Tadpole growth rate was not affected by predator exposition nor correlated with behaviour in this study.

Our results do not exclude the possibility that the observed repeatability arose due to a state–behaviour feedback mechanism related to another state variable. Indeed, weak support is found for state dependence of personality when the state is approximated by singular elements (or effectors) of condition (Rádai et al. 2022). In a multi‐element study, certain measures of body condition affected juvenile and adult gecko exploration but not boldness, while both traits were repeatable (Sakai 2018). Also, multiple mechanisms, that is, different feedbacks, might be at work at a time (Sih et al. 2015).

### 4.3 Adult Behavioural Type Is Related to Body Size Rather Than to Larval Behavioural Phenotype (ad. H2)

Meanwhile, froglet boldness was linked to their body size—time to emergence from cover was shorter in larger frogs—but not to their boldness or activity as tadpoles, corroborating predictions of the adaptive decoupling hypothesis.

Across the several studies on persistence of personalities over metamorphosis, in insects and amphibians, boldness seems to be least often consistent, and activity still less often than exploration (table 1 in Koenig and Ousterhout 2018). That boldness is the first to be decoupled can be expected, as of these traits it is least directly dependent on physiology or neurology, that is, on internal processes, and most on environmental context, including details of the interspecific interaction. Yet, what different authors interpret as activity, boldness or exploration differs from study to study (even if all define them according to Réale et al. 2007). Due to this and to different methodology used, often justified by differences between study systems, comparisons between studies should be made cautiously.

Correlations between life stages may differ between study systems. They should be lower or weaker where there is both, full morphological and full habitat transition, like in our system. Yet, already an earlier study of carry‐over effects between distinctly different life stages showed that personality traits can, indeed, be carried over from larvae in one environment to adult in a completely different environment (odonates; Brodin 2009). Also in amphibians, the pattern of consistency/inconsistency of personality traits over metamorphosis does not follow the partiality/completeness of these transitions (Table 1). Interestingly, some behavioural consistency over metamorphosis has been found in geographically or developmentally homogeneous populations, irrespective of the sample size or time elapsed between trials (Table 1).

Larger froglets emerged earlier from the cover in our study, which could be linked to both their higher boldness or general activity. Froglet body size has been earlier found correlated with total activity, latency to first movement and to resume activity in *R. ridibunda* (Wilson and Krause 2012b)—larger frogs were more active. Also heavier mainland *R. temporaria* froglets were less bold, but had a higher exploration index (Brodin et al. 2013). Similarly, body size predicted inter‐individual differences in exploration behaviour in the southern corroboree frog (Kelleher et al. 2017). Yet, we have not found repeatability in froglet behaviour. Indeed, little consistency or repeatability of time to emergence has been recently raised as an issue. The robustness of the start‐box emergence test, which is a commonly used test, for example, to score boldness in animal personality experiments, has been questioned as it may be sensitive to minor, even unperceived, alterations in procedures (Näslund 2021).

Still, together with the recent findings of Cortazar‐Chinarro et al. (2023), our study suggests moor frog adults may be behaviourally unconstrained by larval experiences, in line with predictions that where pressures faced by larvae are very different from those experienced after metamorphosis, greater plasticity rather than behavioural syndromes are expected.

### 4.4 Summary

Our study confirmed most of the hypotheses. Indeed, the experience of the presence of a predator led to the emergence of a personality in the moor frog tadpoles in one of the two aspects studied, that is, activity, but not boldness. The observed behavioural repeatability disappeared in direct contact with the predator, which could be due to the imposed experimental conditions and the confrontation of potential prey with random predators requiring different defence mechanisms. However, we did not find a relationship between the detected personality of the tadpoles and their body size. Still, such a relationship was found in metamorphosed frogs, in which the time to emergence from cover was inversely related to body size. The behaviour of the frogs was in no way related to their personality as tadpoles, confirming the adaptive decoupling hypothesis. The comparison of the results obtained with literature data encourages the continuation of similar studies, taking into account the specificity of the interactions between larvae of a particular frog species and a particular predator species, and using methods that allow the observation of the real costs of the defence activated by the tadpoles, which would perhaps allow a more reliable verification of hypotheses on the correlation between the observed states and personalities.

## Author Contributions

**Barbara Płaskonka:** data curation (equal), formal analysis (supporting), investigation (equal), writing – original draft (equal), writing – review and editing (equal). **Anna Zaborowska:** conceptualization (equal), investigation (equal), methodology (equal), resources (lead), writing – review and editing (equal). **Andrzej Mikulski:** conceptualization (equal), methodology (supporting), writing – review and editing (equal). **Barbara Pietrzak:** conceptualization (equal), data curation (equal), formal analysis (lead), funding acquisition (lead), investigation (equal), methodology (equal), writing – original draft (equal), writing – review and editing (lead).

## Conflicts of Interest

The authors declare no conflicts of interest.

## Supporting information


Appendix S1.

## Data Availability

Data and code are provided in Appendix S1.

## Appendix 1

### Tadpole Behaviour

*Boldness*. Tadpoles reared in the presence of predator cues (P) spent more time in the open space of the experimental arena than control tadpoles (N) did (Figure A1A, see generalised least squares [GLS] results in Table A1; pairwise Wilcoxon rank sum test, *p* = 0.0088). Rearing conditions (larval environment) had no effect, however, either on the time spent among plants nor on the time spent at the walls of the arena (Table A1, Figure A1B,C). For all three parameters relating to microhabitat choice there was a significant effect of the current environment treatment (*p* < 0.0012, Table A1). In the first, initial phase (I), the tadpoles chose microhabitat differently than after adding experimental medium (II; control medium for N, and medium with predator cue for P treatment). Before adding the medium the tadpoles spent more time in the open, and less time among plants or at the walls than after adding it (Figure A1A–C). There was no effect of adding a live predator (P III) on the overall microhabitat choice (Figure A1A–C, Table A1). Yet, the kind of live predator added (Coleoptera or Anisoptera) affected the time spent in the open area (ANOVA on GLS results, *χ* ^2^ = 8.11, df = 3, *p* = 0.044). Coleoptera‐exposed tadpoles stayed more in the open area than unexposed (N) tadpoles (27% vs. 15% time, *p* = 0.048).

In terms of their activity, control tadpoles (N) reacted differently than tadpoles reared in the presence of predator cues (P) in response to the addition of the experimental medium (I; ANOVA on generalised least squares [GLS] results, significance of interaction term, *χ* ^2^ > 8.8, df = 2, *p* < 0.013, Table A1). After adding the medium they became more active, that is, spent less time immobile and swam faster (both for median and third quartile velocity) than before (Table A1, Figure A1D–F). Both Coleoptera‐ and Anisoptera‐exposed tadpoles stayed more time immobile than the unexposed (*χ* ^2^ = 11.3, df = 3, *p* = 0.01; 66% or 64%, respectively, vs. 53%, *p* < 0.023).

Body length did not explain any of the tadpole behavioural variables, neither as main effects nor in interactions (*p* > 0.2), and were dropped from all the models. Boldness and activity were correlated weakly, that is, tadpoles that stayed more time in the open stayed more time immobile (Pearson's product moment correlation coefficient cor = 0.30, CI: 0.02–0.54; *t* = 2.2, df = 46, *p* = 0.036).

**TABLE A1:** Results of ANOVA performed on best GLS models fitted to boldness and activity data.

Trait	Behavioural parameter/dependent variable	Model/explaining variable	df	*χ* ^2^	*p*
~ Larval environment (N/P) + Current environment (I/II/III)					
Boldness	Time in open	**Larval**	**2**	**38.2**	**< 0.0001**
		**Current**	**1**	**5.75**	**0.0164**
	Time among plants	Larval	1	2.50	0.114
		**Current**	**2**	**26.0**	**< 0.0001**
	Time at walls	Larval	1	0.91	0.34
		**Current**	**2**	**13.6**	**0.0011**
~ Larval environment (N/P) * Current environment (I/II/III)					
Activity	Time immobile	**Larval**	1	0.07	0.93
		**Current**	**2**	**31.5**	**< 0.0001**
		**Interaction**	**2**	**14.1**	**0.0009**
	Median speed	Larval	1	0.14	0.7089
		**Current**	**2**	**40.4**	**< 0.0001**
		**Interaction**	**2**	**8.80**	**0.0122**
	3Q speed	Larval	1	0.00	0.9987
		**Current**	**2**	**86.2**	**< 0.0001**
		**Interaction**	**2**	**20.4**	**< 0.0001**

*Note:* The effects of family of origin and current body size were non‐significant and were dropped from the models. Cases with *p* < 0.05 are bolded.

**TABLE A2:** LMM‐based estimates of repeatability (R), among‐individual variance (Vi) and within‐individual variance (Vr) of tadpole activity throughout the three current environments, their standard errors (SE) and 95% confidence intervals (CI).

Larval environment	Current environment	Parameter	Estimated value	SE	CI	*p*
No predator	I	R	0	0.078	[0, 0.268]	1
		Vi	0	0.003	[0, 0.01]	1
		Vr	0.042	0.008	[0.025, 0.056]	
	II	R	0.059	0.1	[0, 0.322]	0.32
		Vi	0.003	0.006	[0, 0.019]	0.66
		Vr	0.054	0.01	[0.034, 0.074]	
	III	R	0.183	0.129	[0, 0.457]	0.11
		Vi	0.006	0.005	[0, 0.018]	0.55
		Vr	0.027	0.006	[0.017, 0.039]	
Predator	**I**	**R**	**0.422**	**0.13**	[**0.15, 0.636]**	**0.01**
		Vi	0.015	0.007	[0.004, 0.031]	0.5
		Vr	0.021	0.004	[0.013, 0.03]	
	**II**	**R**	**0.264**	**0.134**	**[0, 0.528]**	**0.02**
		Vi	0.011	0.007	[0, 0.027]	0.51
		Vr	0.032	0.006	[0.021, 0.045]	
	III	R	0.155	0.126	[0, 0.426]	0.12
		Vi	0.008	0.007	[0, 0.024]	0.55
		Vr	0.045	0.009	[0.028, 0.065]	

*Note:* Bold values are significant at *p* < 0.05 (based on permutation tests).

**TABLE A3:** LMM‐based estimates of repeatability (R), among‐individual variance (Vi) and intra‐individual variance (Vr) of tadpole boldness throughout the three current environment treatments, their standard errors (SE) and 95% confidence intervals (CI).

Larval environment	Current environment	Parameter	Estimated value	SE	CI	*p*
Predator	I	R	0	0.061	[0, 0.209]	1
		Vi	0	0.004	[0, 0.014]	1
		Vr	0.066	0.012	[0.047, 0.09]	
	II	R	0.014	0.075	[0, 0.246]	0.4
		Vi	0.001	0.005	[0, 0.017]	0.67
		Vr	0.061	0.011	[0.04, 0.081]	
	III	R	0	0.066	[0, 0.213]	1
		Vi	0	0.005	[0, 0.013]	1
		Vr	0.067	0.011	[0.043, 0.086]	
No predator	I	R	0	0.069	[0, 0.214]	1
		Vi	0	0.006	[0, 0.019]	1
		Vr	0.066	0.011	[0.042, 0.089]	
	II	R	0	0.071	[0, 0.008]	1
		Vi	0	0.003	[0, 7.30]	1
		Vr	0.049	0.008	[0.035, 0.065]	
	III	R	0.07	0.09	[0, 0.283]	0.25
		Vi	0.003	0.004	[0, 0.014]	0.66
		Vr	0.04	0.008	[0.028, 0.056]	
